# Impact of Hamstring Graft on Hamstring Peak Torque and Maximum Effective Angle After Anterior Cruciate Ligament Reconstruction: An Exploratory and Preliminary Study

**DOI:** 10.3390/bioengineering12050465

**Published:** 2025-04-28

**Authors:** Ismail Bouzekraoui Alaoui, Ayrton Moiroux-Sahraoui, Jean Mazeas, Georgios Kakavas, Maciej Biały, Maurice Douryang, Florian Forelli

**Affiliations:** 1 Mohammed VI University of Sciences and Health—UM6SS, Casablanca 20270, Morocco; alaoui.ismail.bz@gmail.com; 2 Orthosport Rehab Center, 95330 Domont, France; ayrton.moirouxsahraoui@gmail.com (A.M.-S.); jeanmazeas@gmail.com (J.M.); 3 Orthopaedic Surgery Department, Clinic of Domont, Ramsay Healthcare, @OrthoLab, 95330 Domont, France; 4 Fysiotek Spine and Sports Lab, 11635 Athens, Greece; georgios.kakavas@gmail.com; 5 Department of Physical Education and Sport Science, University of Thessaly, @ErgoMechLab, 42100 Trikala, Greece; 6 Institute of Physiotherapy and Health Sciences, The Jerzy Kukuczka Academy of Physical Education, 40-065 Katowice, Poland; mbfizjoterapia@gmail.com; 7 Functional Diagnostics Laboratory, Sport-Klinika, Scanmed Sport, 44-240 Żory, Poland; 8 Department of Physiotherapy and Physical Medicine, University of Dschang, Dschang P.O. Box 96, Cameroon; douryangmaurice@gmail.com; 9 SFMK Lab, 93380 Pierrefite sur Seine, France; 10 Haute-Ecole Arc Santé, HES-SO University of Applied Sciences and Arts Western Switzerland, 2000 Neuchâtel, Switzerland

**Keywords:** anterior cruciate ligament reconstruction, maximum effective angle, hamstring-to-quadriceps strength ratio, hamstring strength

## Abstract

Purpose: Anterior cruciate ligament reconstruction (ACLR) using the hamstring graft is commonly performed to restore knee stability; however, it induces significant neuromuscular and biomechanical changes, particularly in the hamstring. This study aimed to evaluate the changes in maximum effective angle, hamstring strength, and hamstring-to-quadriceps (H/Q) strength ratio at 3 and 6 months post-ACLR and compare these outcomes to a control group. Methods: This prospective controlled study included 20 ACLR patients and 20 age- and gender-matched controls. Hamstring peak torque, maximum effective angle (MEA), and the H/Q ratio were assessed using isokinetic dynamometry at 60°/s. The ACLR group was evaluated postoperatively at 3 and 6 months, while the control group underwent a single evaluation. Results: At 3 and 6 months, the ACLR group exhibited significantly lower MEA (26.3° ± 8.2 and 28.2° ± 9.4) compared to the control group (36.4° ± 12.0; *p* < 0.01). Hamstring peak torque and H/Q ratios were also lower in the ACLR group but showed slight improvements over time. The H/Q ratio increased significantly between 3 and 6 months (51% to 56%; *p* = 0.041). Conclusion: The use of hamstring graft in ACLR leads to persistent MEA and strength deficits despite rehabilitation. Advanced, targeted rehabilitation protocols are essential to address these deficits, optimize recovery, and reduce the risk of reinjury.

## 1. Introduction

The anterior cruciate ligament (ACL) is one of the most injured structures in the knee, particularly among athletes. ACL tears account for approximately 50% of all knee injuries, with an increasing incidence attributed to the rising intensity and diversity of sports participation [1,2]. However, this injury is not confined to athletes; sedentary individuals are also at risk, often due to traumatic events or degenerative changes [3,4,5]. ACL injuries can result in substantial physical, psychological, and financial burdens due to their significant functional impact, the requirement for surgical intervention, and prolonged rehabilitation periods [1].

Anterior cruciate ligament reconstruction (ACLR) using autografts harvested from the patient’s hamstring tendons, specifically the semitendinosus and gracilis, is the gold standard for treatment in young and active individuals. While effective at restoring knee stability, this approach induces significant morphological and neuromuscular changes in the harvested muscles [6]. Among these changes, alterations in the maximum effective angle (MEA) of the hamstring—defined as the joint angle at which maximum torque is produced—are of particular concern [7,8,9]. The MEA serves as a critical biomechanical indicator of hamstring efficiency and stability, directly influencing lower limb functionality and injury risk [10,11].

Hamstring function plays a pivotal role in protecting the knee during high-demand activities, such as sprinting, pivoting, and landing [12,13,14]. The altered biomechanical properties of the hamstrings after ACLR may predispose individuals to secondary injuries, particularly hamstring strain injuries, which are among the most common muscle injuries in sports [15]. Retrospective studies have consistently shown that individuals with a history of hamstring strain injuries exhibit higher MEA values (more flexed knee positions) compared to uninjured controls [10,11]. Despite the clinical relevance, the extent to which ACLR influences MEA and its potential long-term implications remain underexplored.

Postoperative rehabilitation aims to restore muscle strength, neuromuscular control, and functional stability [16,17,18]. However, deficits in hamstring strength have been observed even years after ACLR, with reported reductions of up to 30% compared to the contralateral limb [19,20]. These deficits, compounded by neuromotor changes induced by tendon harvesting, are likely to affect the MEA, potentially increasing the risk of reinjury or secondary musculoskeletal complications [21,22]. Furthermore, the relationship between MEA changes and muscle strength recovery during rehabilitation is poorly understood, necessitating further investigation [6].

Studies have also highlighted the importance of the hamstring-to-quadriceps strength ratio in reducing the risk of lower limb injuries [23,24]. Ratios below 60% are considered suboptimal, with a higher risk of knee instability and hamstring strain injuries [25,26]. Additionally, hamstring activity during dynamic knee flexion varies significantly depending on the MEA, reinforcing its role as a predictor of functional recovery and injury risk [27]. Understanding how ACLR affects MEA at different rehabilitation stages could provide critical insights into optimizing postoperative protocols [28,29,30].

This study aims to evaluate the changes in the MEA of the hamstring at 3 and 6 months following ACLR using the hamstring graft. By comparing postoperative patients to a control group of uninjured individuals, this research seeks to identify the extent and nature of MEA alterations, their relationship with muscle strength recovery, and the potential implications for injury prevention. Ultimately, these findings aim to inform evidence-based rehabilitation strategies to enhance patient outcomes and mitigate the risk of secondary injuries.

## 2. Methods

### 2.1. Study Design

This study was a prospective controlled trial designed to evaluate changes in the MEA of hamstrings following ACLR using a hamstring graft. Data were collected from two groups: (1) a test group comprising individuals who underwent ACLR and (2) a control group of uninjured participants. The test group was assessed postoperatively at 3 and 6 months, while the control group was assessed once. This study adhered to ethical guidelines, with prior approval from the local ethics committee and written informed consent obtained from all participants according to the Declaration of Helsinki, World Medical Association, 2013.

### 2.2. Randomization

This study was conducted without randomization due to ethical and logistical constraints associated with assigning surgical interventions in real-world clinical settings. As this was an exploratory and preliminary investigation, our primary aim was to assess postoperative changes in hamstring biomechanics under typical clinical conditions. To mitigate selection bias, we employed strict inclusion and exclusion criteria and matched participants in the control group to the ACLR group based on age, sex, BMI, and activity level. Baseline comparability between groups was confirmed through statistical analysis. Nonetheless, we acknowledge that random allocation would enhance internal validity, and we recommend that future studies adopt randomized controlled designs to validate and expand upon these findings.

### 2.3. Participants

Participants were recruited from a private orthopedic clinic and outpatient physiotherapy centers. Inclusion criteria for the test group were as follows: (1) age between 18 and 35 years, (2) primary ACLR performed using hamstring graft, and (3) no other lower limb injuries or neurological impairments. Exclusion criteria included revision ACLR, any congenital knee deformities, or ongoing joint effusion.

The control group consisted of age- and gender-matched healthy individuals with no history of lower limb injuries or surgeries. All participants in both groups were physically active (engaged in moderate exercise ≥3 times per week).

A total of 40 participants (20 in each group) were included in this study. Demographic data, including age, BMI, and gender, were recorded. Baseline comparability between groups was confirmed, ensuring no statistically significant differences in these parameters [20,31,32].

The contralateral limb was not used as a control in this study, as previous research indicates that it may undergo compensatory neuromuscular changes following ACL injury and reconstruction. Instead, a separate control group of healthy individuals was included to provide a more accurate baseline for comparison.

### 2.4. Sample Size Calculation

The sample size calculation was performed using G*Power version 3.1.9.7, conducting a power analysis for an independent two-group comparison. Considering a large effect size (Cohen’s d = 0.8), a significance level of 5% (α = 0.05), and a statistical power of 80% (1 − β = 0.80), it was estimated that a minimum of 26 participants per group (52 in total) would be required to detect a significant difference between the groups.

### 2.5. Rehabilitation Protocol

The rehabilitation program for the test group followed evidence-based guidelines, emphasizing progressive load-bearing, neuromuscular training, and functional strengthening [17]. The key phases were as follows:

Weeks 0–6 (early recovery): Focused on pain and swelling management, passive range of motion restoration, and activation of the quadriceps and hamstrings. Patients were encouraged to achieve full knee extension within 2 weeks.

Weeks 7–12 (strength building): Progressive resistance training was introduced, targeting both concentric and eccentric strength of the quadriceps and hamstrings. Balance and proprioceptive exercises were incorporated.

Weeks 13–24 (advanced strengthening): High-intensity exercises, including plyometrics and sport-specific drills, were introduced to restore pre-injury performance levels. Return-to-sport readiness was evaluated using functional tests such as single-leg hop tests and isokinetic strength testing [33].

Participants received individualized physiotherapy sessions three times per week, with adherence monitored through attendance logs.

### 2.6. Assessment Protocol

Maximum effective angle measurements were obtained using an isokinetic dynamometer (Biodex System 4, Biodex Medical Systems, Shirley, NY, USA). Participants were seated with their hip flexed at 90° and their knee stabilized to prevent extraneous movements. The isokinetic testing involved four repetitions of maximal knee flexion and extension at a velocity of 60°/s, as this speed is considered optimal for assessing muscle strength and torque [27].

The maximum effective angle was defined as the joint angle at which the hamstring peak torque was generated during the knee flexion phase. Measurements were conducted bilaterally for the test group (operated and contralateral limbs) and unilaterally for the control group. To ensure reliability, each participant performed a familiarization session prior to testing, and all assessments were conducted by the same investigator [25,26].

Secondary outcomes included the hamstring peak torque value (in Newton-meters) and the hamstring-to-quadriceps strength ratio, which were calculated and compared across groups.

The test group was evaluated at two distinct postoperative time points: at 3 months, with an average interval of 3.37 ± 0.42 months following surgery, and at 6 months, with an average interval of 6.21 ± 0.40 months following surgery.

### 2.7. Statistical Analysis

Statistical analyses were performed using SPSS software (v26.0; IBM, Armonk, NY, USA). The normality of the data was assessed using the Shapiro–Wilk test. Between-group comparisons of MEA values were conducted using the Mann–Whitney U test, as the data were not normally distributed. Within-group comparisons (e.g., 3- vs. 6-month assessments in the test group) were performed using paired t-tests for normally distributed data or Wilcoxon signed-rank tests for non-parametric data.

The normality of variables used in correlation analyses (MEA and hamstring peak torque) was assessed using the Shapiro–Wilk test. If normality was not confirmed, Spearman’s rank correlation was used instead of Pearson’s.

The correlation between MEA and hamstring peak torque was evaluated using Pearson’s correlation coefficient, with significance set at *p* < 0.05. Descriptive statistics, including means and standard deviations, were reported for all variables.

## 3. Results

### 3.1. Participant Characteristics

A total of 40 participants were recruited and evenly distributed into two groups: the test group (n = 20) and the control group (n = 20). No statistically significant differences were observed between the groups in terms of demographic or baseline characteristics, ensuring comparability.

Test group: The mean age was 26.4 years (±8.1), and the mean BMI was 23.5 kg/m^2^ (±3.3). This group included 10 males and 10 females.

Control group: The mean age was 27.0 years (±5.5), and the mean BMI was 24.0 kg/m^2^ (±3.6). This group included 10 males and 10 females.

The proportion of left (45%) and right (55%) knees tested was balanced across both groups, and no significant differences were detected (*p* > 0.05). The participants’ characteristics are summarized in Table 1.

### 3.2. Maximum Effective Angle

#### 3.2.1. Comparison Between Test and Control Groups

At 3 months post-surgery, (Table 2) the test group exhibited a significantly lower mean MEA (26.3° ± 8.2) compared to the control group (36.4° ± 12.0; *p* = 0.002). At 6 months, (Table 2) the test group showed a slight increase in the mean MEA to 28.2° ± 9.4, which remained significantly lower than the control group (*p* = 0.037).

#### 3.2.2. Within-Group Comparison (Test Group)

In the test group, the MEA increased marginally from 3 months (26.3° ± 8.2) to 6 months (28.2° ± 9.4). However, this change did not reach statistical significance (*p* = 0.089), indicating that the MEA showed limited improvement over time despite ongoing rehabilitation (Table 3).

### 3.3. Hamstring Peak Torque

#### 3.3.1. Comparison Between Test and Control Groups

At 3 months post-surgery, the mean hamstring peak torque in the test group was 55.4 Nm ± 34.5, compared to 73.9 Nm ± 36.7 in the control group (Table 4). Although the test group produced a lower peak torque, the difference was not statistically significant (*p* = 0.081). At 6 months, the mean hamstring peak torque in the test group increased slightly to 80.3 Nm ± 30.6, but it remained lower than the control group (*p* = 0.067) (Table 4).

#### 3.3.2. Within-Group Comparison (Test Group)

The test group showed a numerical increase in hamstring peak torque (Table 5) from 3 months (55.4 Nm ± 34.5) to 6 months (80.3 Nm ± 30.6); this difference was statistically significant (*p* = 0.0094).

### 3.4. Hamstring-to-Quadriceps Strength Ratio

#### 3.4.1. Comparison Between Test and Control Groups

At 3 months, the mean H/Q strength ratio (Table 6) in the test group was 51% ± 12, significantly lower than the control group (63% ± 14; *p* = 0.027). At 6 months, the test group’s H/Q ratio increased to 56% ± 13, but it remained significantly lower than the control group (*p* = 0.034).

#### 3.4.2. Within-Group Comparison (Test Group)

The H/Q strength ratio (Table 7) in the test group showed a statistically significant increase between 3 months (51% ± 12) and 6 months (56% ± 13; *p* = 0.041), reflecting a moderate improvement in hamstring strength relative to quadriceps strength over time.

#### 3.4.3. Correlation Between MEA and Hamstring Peak Torque

**Figure 1:**
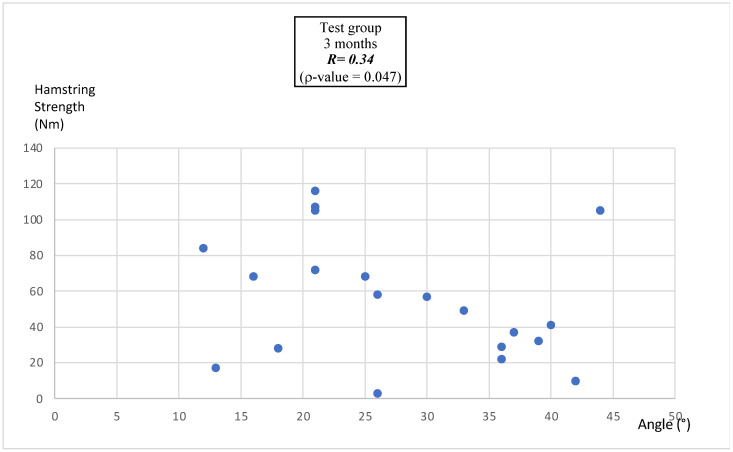
Pearson correlation between MEA and hamstring peak torque at 3 months after ACLR in the test group.

At 3 months post-surgery (Figure 1), a weak but statistically significant positive Pearson’s correlation was observed between the MEA and hamstring peak torque (r = 0.34, *p* = 0.047).

**Figure 2:**
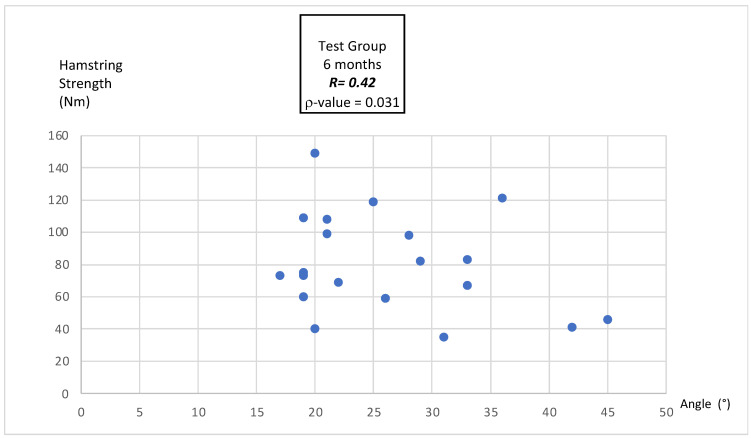
Pearson correlation between MEA and hamstring peak torque at 6 months after ACLR in the test group.

At 6 months post-surgery, Pearson’s correlation was slightly stronger, with a positive and statistically significant relationship (r = 0.42, *p* = 0.031), indicating a modest improvement in the association as rehabilitation progressed (Figure 2).

## 4. Discussion

The purpose of this study was to evaluate the changes in the MEA of the hamstring at 3 and 6 months following ACLR using a hamstring graft and to assess the associated muscle strength deficits and H/Q strength ratios. The results demonstrated that MEA remains significantly lower in the test group compared to the control group at both times, with minimal within-group improvement. Additionally, peak torque and the H/Q ratio showed persistent deficits in the test group, although modest improvements were noted over time.

### 4.1. Maximum Effective Angle

The significantly lower MEA observed in the test group at 3 and 6 months post-surgery aligns with previous findings that ACLR involving autograft harvesting from the hamstring tendons induces neuromuscular alterations and morphological changes in these muscles [6,34]. The reduction in the MEA reflects a shift in the joint angle at which peak torque is generated, suggesting compromised mechanical efficiency of the hamstring. This phenomenon can be attributed to several factors. First, autograft harvesting from the semitendinosus and gracilis tendons disrupts their normal architecture, resulting in reduced muscle volume, cross-sectional area, and altered tendon stiffness [6,35]. Second, neuromotor deficits, including altered motor unit recruitment patterns and proprioceptive feedback, may contribute to the inability of the knee flexors to generate maximum torque at their pre-injury angles [36,37,38]. These deficits are particularly evident during concentric contractions, as observed in this study, where isokinetic dynamometry is used at 60°/s—a velocity widely regarded as reliable for assessing MEA [10,11]. The limited improvement in MEA overtime within the test group suggests that standard rehabilitation protocols may not adequately address these deficits. Current rehabilitation programs often focus on restoring overall strength and functional stability, but they may neglect the specific biomechanical alterations caused by autograft harvesting [17]. Targeted interventions, such as eccentric strengthening and neuromuscular rehabilitation, may be necessary to optimize MEA recovery and reduce the risk of secondary injuries [29].

### 4.2. Hamstring Peak Torque Deficits

The persistent deficits in hamstring peak torque observed in the test group at both 3 and 6 months post-surgery are consistent with earlier studies reporting prolonged strength impairments following ACLR [19,20]. Although the test group demonstrates a non-significant increase in torque between 3 and 6 months, their values remain below those of the control group, highlighting the long-lasting impact of ACLR on hamstring strength. One plausible explanation for these deficits is the incomplete recovery of the semitendinosus and gracilis muscles post-harvesting. Studies using MRI and electromyography have shown reduced muscle volume and activity levels in these muscles up to two years post-surgery [6,35]. Additionally, compensatory hypertrophy of the biceps femoris may occur, but this adaptation is unlikely to fully restore the functional capacity of the hamstrings as a whole [34]. This suggests that despite strength improvements during rehabilitation, full recovery of torque generation may remain elusive without targeted interventions.

### 4.3. Hamstring-to-Quadriceps Strength Ratio

The significantly lower H/Q strength ratio observed in the test group compared to the control group at both points reinforces the importance of addressing hamstring strength deficits during rehabilitation. A reduced H/Q ratio is a well-documented risk factor for lower limb injuries, particularly hamstring strain injuries and ACL graft failure [24,26,27]. The modest but significant improvement in the H/Q ratio within the test group between 3 and 6 months suggests that standard rehabilitation protocols can partially restore balance between muscle groups. However, the ratio remains below the 60–75% threshold considered optimal for knee stability and injury prevention [2,20,23]. The delayed recovery of the H/Q ratio may also reflect altered biomechanics following ACLR. Autograft harvesting can lead to an imbalance in force production between knee flexors and extensors, with the quadriceps often recovering more rapidly than the hamstrings [3,39,40]. This imbalance underscores the need for progressive resistance training programs that specifically target hamstring strength, particularly eccentric strengthening, which has been shown to be effective in restoring the H/Q ratio and reducing injury risk [17,27].

### 4.4. Correlation Between MEA and Hamstring Peak Torque

The results demonstrate a weak but statistically significant positive correlation between the MEA and hamstring peak torque at both 3 months (*r* = 0.34, *p* = 0.047) and 6 months (*r* = 0.42, *p* = 0.031) post-surgery. The slightly stronger correlation at 6 months suggests that the relationship between the angle of peak torque generation and hamstring strength improves as rehabilitation progresses. This progression may reflect neuromuscular adaptations and biomechanical improvements in the hamstrings following surgery and targeted rehabilitation. These findings are consistent with previous studies indicating that neuromuscular recovery plays a crucial role in restoring functional strength and mechanical efficiency post-ACLR [6,20,32,41]. Specifically, the correlation between the MEA and peak torque may highlight the gradual improvement in hamstring performance, including better recruitment of motor units and recovery of muscle-tendon dynamics during the rehabilitation process [34,36]. Despite the improvement observed over time, the modest correlation coefficients (*r* = 0.34 and *r* = 0.42) suggest that other factors beyond the MEA influence hamstring peak torque. For instance, muscle architecture changes, such as reduced cross-sectional area and tendon stiffness following graft harvesting, likely contribute to torque deficits [35,42,43,44,45]. Additionally, neuromotor impairments, including altered motor unit recruitment and proprioceptive deficits, may further impact the efficiency of torque generation [17,42,43,46,47]. Clinically, these results emphasize the importance of monitoring the MEA and its relationship with hamstring strength as part of rehabilitation. Improvements in this correlation may serve as an indirect marker of hamstring recovery and neuromuscular reorganization. However, additional research is needed to fully understand the interplay between the MEA and torque production and to explore whether interventions targeting specific joint angles during rehabilitation can enhance this relationship [48,49,50].

## 5. Limits

This study provides valuable insights into neuromuscular and biomechanical changes following ACLR using a hamstring graft; however, several limitations should be acknowledged. The relatively small sample size and homogeneity of participants (age range of 18–46 years, physically active individuals) may limit generalizability to broader populations, including older adults and elite athletes. Additionally, the absence of randomization may have introduced selection bias and limited the internal validity of our findings. While we used strict inclusion criteria and group matching to reduce this risk, future studies should consider randomized controlled designs to strengthen causal inferences. The short follow-up period of 6 months captures early rehabilitation outcomes but does not reflect long-term recovery or persistent deficits. Finally, the 6-month follow-up period limits our ability to assess long-term outcomes, such as sustained MEA recovery and full functional reintegration. Future studies should incorporate 12-month or longer follow-up intervals to better capture the durability of rehabilitation effects. Furthermore, although our power analysis recommended a minimum of 52 participants, we included only 40 due to recruitment constraints and strict eligibility criteria, which may have reduced the statistical power and generalizability of the findings. Additionally, this study focused solely on concentric hamstring strength, omitting assessments of eccentric or isometric strength, which are crucial for knee stability and injury prevention [51,52]. Future studies should include all contraction types to provide a more complete neuromuscular profile. Functional outcomes, such as return-to-sport readiness or patient-reported measures, were not assessed, limiting the clinical interpretation of the biomechanical findings. Although a standardized rehabilitation protocol was used, individual variations in adherence and access to resources could have influenced outcomes. Rehabilitation adherence was monitored using physiotherapy attendance logs, which, while practical, may not fully reflect true patient engagement or unsupervised exercise compliance. Future studies should consider integrating objective tracking tools—such as digital apps or wearable sensors—to enhance adherence monitoring and data accuracy. Moreover, the absence of imaging data, such as MRI or ultrasound, prevents a direct correlation between the observed deficits and structural changes in the hamstrings or graft site. Future research should address these limitations by including larger, more diverse populations, longer follow-up periods, comprehensive strength assessments, functional outcomes, and imaging-based evaluations to better inform rehabilitation strategies and optimize patient outcomes.

## 6. Conclusions

This study demonstrates persistent deficits in the MEA, hamstring strength, and the hamstring-to-quadriceps ratio following ACLR using a hamstring graft, despite modest improvements between 3 and 6 months. These findings highlight the need for advanced rehabilitation strategies, including eccentric strengthening and neuromuscular re-education, to address specific biomechanical and neuromuscular challenges. Future research should focus on long-term recovery, functional outcomes, and patient-centered approaches to optimize rehabilitation and reduce reinjury risk.

## Figures and Tables

**Table 1:** Participants’ characteristics.

Characteristic	Control Group (n = 20)	Test Group at 3 Months (n = 20)	Test Group at 6 Months (n = 20)	*p*-Value
Age (years)	27 (±5.46)	26.25 (±8.14)	26.50 (±8.14)	0.64
BMI (kg/m^2^)	24.05 (±3.57)	23.50 (±3.30)	23.50 (±3.30)	0.53
Sex (Male/Female)	Male: 10/Female: 10	Male: 10/ Female: 10	Male: 10/ Female: 10	0.89
Side Tested (Left/Right)	Left: 9/Right: 11	Left: 9/Right: 11	Left: 9/Right: 11	0.78
Interval Between Surgery and Assessment (Months)	Ø	3.37 (±0.42)	6.21 (±0.40)	Ø

**Table 2:** Comparison of MEA between groups.

Time Point	Control Group (n = 20)	Test Group (n = 20)	*p*-Value
MEA (°) 3 Months Post-Surgery	36.4 ± 12.0	26.3 ± 8.2	0.0019
MEA (°) 6 Months Post-Surgery	36.4 ± 12.0	28.2° ± 9.4	0.037

Note: MEA, maximum effective angle.

**Table 3:** Intra-group comparison of MEA.

Time Point	Test Group at 3 Months (n = 20)	Test Group at 6 Months (n = 20)	*p*-Value
MEA (°)	26.3 ± 8.2	28.2 ± 9.4	0.089

Note: MEA, maximum effective angle.

**Table 4:** Comparison of hamstring peak torque between groups.

Time Point	Control Group (n = 20)	Test Group (n = 20)	*p*-Value
HPT (Nm) 3 Months Post-Surgery	73.9 ± 36.7	55.4 ± 34.5	0.081
HPT (Nm) 6 Months Post-Surgery	73.9 ± 36.7	80.3 ± 30.6	0.067

Note: HPT, hamstring peak torque.

**Table 5:** Intra-group comparison of hamstring peak torque.

Time Point	Test Group at 3 Months (n = 20)	Test Group at 6 Months (n = 20)	*p*-Value
HPT (Nm)	55.4 ± 34.5	80.3 ± 30.6	0.0094

Note: HPT, hamstring peak torque.

**Table 6:** Hamstring-to-quadriceps strength ratio.

Time Point	Control Group (n = 20)	Test Group (n = 20)	*p*-Value
H/Q Ratio (%) 3 Months Post-Surgery	63 ± 14	51 ± 12	0.027
H/Q Ratio (%) 6 Months Post-Surgery	63 ± 14	56 ± 13	0.034

Note: H/Q, hamstring-to-quadriceps.

**Table 7:** Intra-group comparison of hamstring-to-quadriceps strength ratio.

Time Point	Test Group at 3 Months (n = 20)	Test Group at 6 Months (n = 20)	*p*-Value
H/Q Ratio (%)	51 ± 12	56 ± 13	0.041

Note: H/Q, hamstring-to-quadriceps.

## Data Availability

The data presented in this study are available on request from the corresponding author.
